# Studying Different Operating Conditions on Reverse Osmosis Performance in the Treatment of Wastewater Containing Nickel (II) Ions

**DOI:** 10.3390/membranes12111163

**Published:** 2022-11-18

**Authors:** Ihab Shigidi, Ramzi H. Harharah, Ghassan M. T. Abdalla, Abubakar Elkhaleefa, Norah S. Alsaiari, Hamed N. Harharah, Abdelfattah Amari, Mohamed G. Hassan

**Affiliations:** 1 Department of Chemical Engineering, College of Engineering, King Khalid University, Abha 61421, Saudi Arabia; 2 Department of Electrical and Electronic Engineering, Faculty of Engineering, University of Khartoum, Khartoum P.O. Box 10179, Sudan; 3 Department of Chemistry, College of Science, Princess Nourah bint Abdulrahman University, P.O. Box 84428, Riyadh 11671, Saudi Arabia; 4 Research Laboratory of Processes, Energetics, Environment and Electrical Systems, National School of Engineers, Gabes University, Gabes 6072, Tunisia; 5 Chemical Engineering, Faculty of Engineering and Physical Sciences, The University of Southampton Highfield Campus, Southampton SO17 1BJ, UK

**Keywords:** reverse osmosis, nickel (II) removal, industrial wastewater, temperature correction factor, mathematical models

## Abstract

The reverse osmosis performance in removing nickel ions from artificial wastewater was experimentally and mathematically assessed. The impact of temperature, pressure, feed concentration, and feed flow rate on the permeate flux and Ni (II) rejection % were studied. Experiments were conducted using a SEPA CF042 Membrane Test Skid—TFC BW30XFR with applied pressures of 10, 20, 30, and 40 bar and feed concentrations of 25, 50, 100, and 150 ppm with varying operating temperatures of 25, 35, and 45 °C, while the feed flow rate was changed between 2, 3.2, and 4.4 L/min. The permeate flux and the Ni (II) removal % were directly proportional to the feed temperature and operating pressure, but inversely proportional to the feed concentration, where the permeate flux increased by 49% when the temperature was raised from 25 to 45 °C, while the Ni (II) removal % slightly increased by 4%. In addition, the permeate flux increased by 188% and the Ni (II) removal % increased to 95.19% when the pressure was raised from 10 to 40 bar. The feed flow rate, on the other hand, had a negligible influence on the permeate flux and Ni (II) removal %. The temperature correction factor (TCF) was determined to be directly proportional to the feed temperature, but inversely proportional to the operating pressure; nevertheless, the TCF was unaffected either by the feed flow rate or the feed concentration. Based on the experimental data, mathematical models were generated for both the permeate flux and nickel removal %. The results showed that both models matched the experimental data well.

## 1. Introduction

Water is becoming increasingly polluted as a result of industrial and urban expansions. Substantial contaminants are discharged globally, leading to huge deteriorations of the environment because of their effects. Heavy metal water pollution is a very destructive type of pollution, as heavy metals are non-degradable and poisonous agents that accumulate in nature with time and pose challenges to human health and the environment [1].

Nickel, as a heavy metal, has the following chemical properties: an atomic weight of 58.69 g/mol and a density of 8.91 g/cm^3^, with an impact on the ecosystem as a major industrially discharged contaminant [2]. Many industries contribute to the presence of nickel (II) in wastewater, including galvanization, paint, and powder manufacturing; battery processing; metal refining; and superphosphate fertilizer production [1]. Nickel exposure over time causes chronic bronchitis, reduced lung function, lung cancer, and nasal sinus cancer. The US Environmental Protection Agency (USEPA) has stated that the highest acceptable contamination level (MCL) for nickel is 0.2 mg/L [3].

Nickel must be removed from industrially discharged water before it is released, and this can be achieved using a variety of treatment techniques, such as chemical precipitation [4,5], flotation [6], coagulation–flocculation [7,8], ion exchange [9,10], electrochemical treatment [11,12], adsorption [13,14,15], and membrane filtration [16,17,18,19,20,21,22,23,24,25,26,27]. Every one of these techniques has its own set of advantages and disadvantages.

Membrane technology exhibits advantages over other methods as an effective wastewater treatment procedure. It is a compact system with economic viability that can operate at different scales [28,29]. A membrane’s ability to remove heavy metal ions is influenced by many factors, including the type of membrane, design parameters, and operating conditions [30,31]. Reverse osmosis membranes (ROMs) are applied in a variety of separation processes such as saltwater desalination, wastewater treatment, and many other industrial waste treatment processes, as they have the ability to provide high contaminant removal percentages [32].

A variety of publications over time have discussed the rejection of nickel (II) by ROMs via alternating conditions such as the applied pressure, inlet temperatures, flow rate, pH, and the inlet concentrations [23,24,25,26,27]. Table 1 highlights the number of studies where nickel (II) ions were rejected by different membrane techniques.

The feed water temperature has an impact on the performance of ROMs [33]. The flow of permeate increases with increasing temperature, as it decreases the solution’s viscosity and increases the membrane surface diffusivity [34,35].

This work aims to investigate the reverse osmosis rejection of nickel ions from artificial wastewater under different operating temperatures, pressures, flow rates, and feed concentrations. The temperature correction factor (TCF) was also investigated and the parameters that influence it were specified. Mathematical models were developed based on the experimental data for nickel removal percentages and permeate flux.

## 2. Methods

### 2.1. Studied Wastewater Samples

A 4000 mg/L nickel stock solution was prepared from nickel nitrate hexahydrate salt (Ni(NO_3_)_2_· 6H_2_O) acquired from Lobachemie Co. (Mumbai, India). From this stock, diluted nickel solutions with concentrations of 25, 50, 100, and 150 mg/L were prepared.

### 2.2. ROM Setup

**Figure 1:**
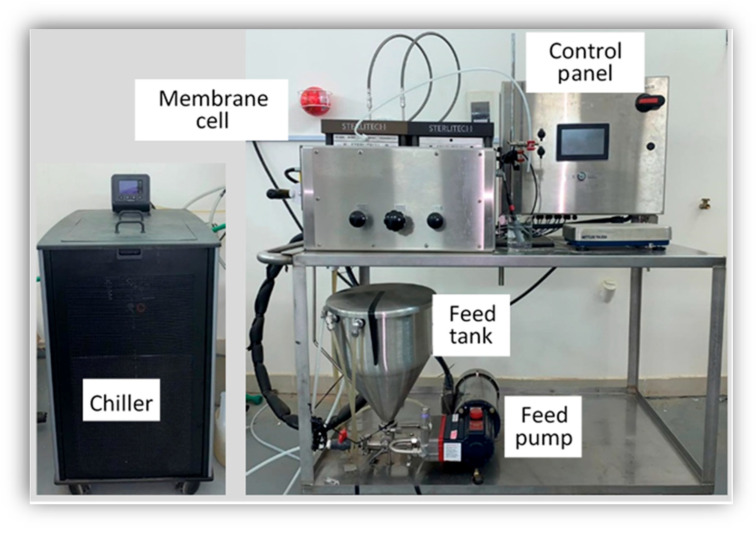
SEPA Cross-Flow Membrane Rig.

Experiments were carried out using a Sterlitech Co. (Auburn, Al, USA) SEPA CF042 Membrane Test Skid, presented in Figure 1 with the scheme for the crossflow filtration ROM processes. A Dow Polyamide TFC BW30XFR flat sheet membrane was utilized under an acidity range of 2–11 and 100 Da MWCO with a 140 cm^2^ membrane area.

### 2.3. Test Method

The experiments were conducted under operating pressures of 10, 20, 30, and 40 bar and feed concentrations of 25, 50, 100, and 150 ppm with a varying operating temperature of 25, 35, and 45 °C, while the feed flow rate was changed between 2, 3.2, and 4.4 L/min. Filtered water was gathered and weighed every 10 min for acquiring the permeate mass, which was used to calculate the permeate flux and metal ion removal. To keep the feed concentration stable, the permeate and retentate water were continually returned to the feed tank. For each new feed concentration, a new membrane sheet was used. Before testing a different concentration, the apparatus was cleaned for at least 10 min with distilled water. A total dissolved solids (TDS) meter (VSTAR20, Thermo-Scientific, Waltham, MA, USA) was used to analyze the produced water samples.

### 2.4. Test Calculations

The filtrate flux (permeate) was calculated according to Equation (1):

(1) $$Permeate\;Flux\;\left(J\right)=\frac{Permeate\;mass\;\left(kg\right)}{\;Process\;time\;\left(h\right)\times Membrane\;area\;\left(m^{2}\right)\;}$$

The nickel ion removal was calculated using Equation (2):

(2) $$R\%=\left[1-\frac{\;Permeate\;Concentration}{\;Feed\;Concentration}\right]\times 100\%$$

## 3. Results and Discussion

### 3.1. Feed Temperature Effect

**Figure 2:**
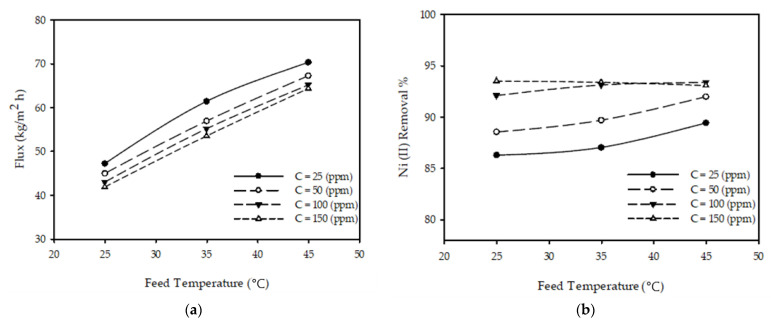
Temperature impact on (**a**) permeate flux and (**b**) Ni (II) removal % for various inlet concentrations with a pressure of 10 bar and a constant feed flow rate of 3.2 L/min.

Figure 2a demonstrates the changes noted on the permeate flux when alternating the operating temperatures while maintaining a constant inlet flow rate and applied pressure. The results revealed that raising the temperature to 40 °C with a constant pressure of 10 bar, a feed flow rate of 3.2 L/min, and an initial concentration of 50 ppm led to the permeate flux increasing from 45.01 to 67.28 kg/m^2^ h, representing an increase of 49% (the permeate was observed to double with every 1 °C increase) due to changes in the physical properties of the polymeric membrane and a reduction in the solution’s viscosity, as observed in various literature [28,36,37,38,39]. This affirmed that as the temperature increases, the permeate flux increases consequently.

The impacts of the inlet temperature on the rejection % of nickel ions at various concentrations of the feed with a constant feed flow rate and pressure are presented in Figure 2b. It was thus noted from the obtained results that when the temperature increased from 25 °C by 20 with the rest of parameters kept constant, the removal % of nickel ions increased to 91.98% from 88.55, as observed by the authors of [28,36,39], leading to the conclusion that an increase in temperature causes the removal % of metal ions to increase.

### 3.2. Operating Pressure Effect

**Figure 3:**
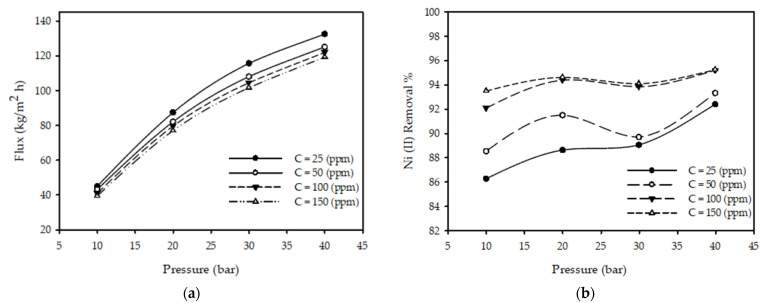
Effect of pressure on (**a**) permeate flux and (**b**) Ni (II) removal % under alternating feed concentrations with a fixed inlet feed flow rate of 3.2 L/min and a feed temperature of 25 °C.

Figure 3a demonstrates the effect of the applied pressure on the permeate under different feed concentrations when the feed temperature and feed flow rate are fixed. It was observed that when the pressure increased by 30 bar from 10 to 40 bar with a constant T of 25 °C, feed flow rate of 3.2 L/min, and initial concentration of 100 ppm, the permeate flux increased by 188% from 43.03 to 123.91 (kg/m^2^ h) and increased by 94% when the pressure increased slightly by 10 bar from 10, by 29.6% when the applied pressure increased from 20 to 30 bar, and by 14.84% when the pressure increased from 30 to 40 bar for all tested concentrations due to the pressure acting as a driving force. Hence, the amount of solute crossing the membrane increased as described in [27,35,37,39,40], which state that the filtrate flow rate increases as the pressure increases.

Figure 3b reports the impacts of the operating pressure on the nickel ion rejection % at various inlet concentrations whilst fixing the temperature and feed flow rate, concluding that an increase in pressure from 10 to 40 bar under the constant parameters T = 25 °C, *C_F_* = 100 ppm, and *Q_F_* = 3.2 L/min resulted in the nickel ion rejection % increasing from 92.09 to 95.19%. This was due to a greater polarization of metal ions on the membrane surface and a concentration decrease in the permeate, as conveyed in [27,35,37,39,40] and confirming that pressure increases cause an increase in the removal % of metal ions.

### 3.3. Feed Concentration Effect

**Figure 4:**
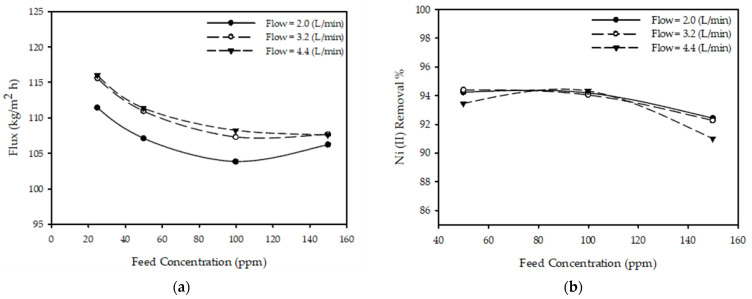
Feed concentration impact on (**a**) permeate flux and (**b**) Ni (II) removal % for feed flow rates with a constant pressure of 20 bar and a temperature of 45 °C.

The effects of maintaining a constant pressure and inlet flow rate temperature whilst varying the feed concentration on the permeate flux for different feed flow rates are presented in Figure 4a, demonstrating that changing the feed concentration from 25 to 150 ppm at a constant T = 45 °C, P = 20 bar, and *Q_F_* = 3.2 L/min resulted in a decrease in the filtrate flux by 7.4% from 115.57 to 107.65 kg/m^2^ h. This can be attributed to the increase in the filtration resistance towards the filtrate across the membrane and the concentration polarization on the membrane surface. This matches the observations that the permeate flux decreases with increasing feed filtrate concentrations, as reported by the authors of [27,35,39].

Figure 4b reports the results of fixing T = 45 °C, P = 20 bar, and *Q_F_* = 3.2 L/min while varying the feed concentration from 50 to 150, and the effects of the feed concentration on the nickel ion rejection % at different inlet flow rates. The nickel ion removal % decreased from 94.39 to 92.27% due to an increase in the concentration polarization on the membrane surface, leading to the observation of a decrease in the removal % of metal ions with increasing feed concentrations. These findings are aligned with those in the literature [27,35,37,39].

### 3.4. Feed Flow Rate Effect

**Figure 5:**
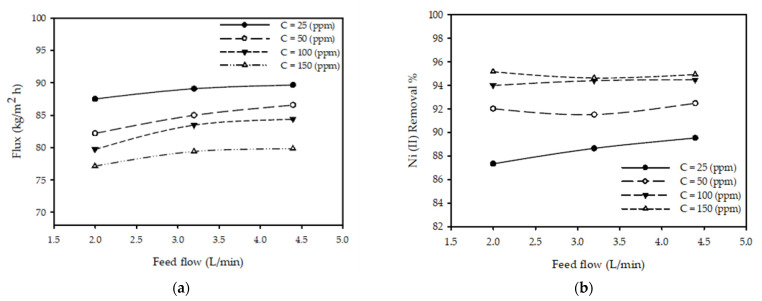
Flow rate impact on (**a**) permeate flux and (**b**) Ni (II) removal % at alternating inlet concentrations with a pressure of 20 bar and a feed temperature of 25 °C.

Figure 5a displays the effect of fixing the pressure, feed concentration, and temperature (T = 25 °C, *C_F_* = 100 ppm, and P = 20 bar). The results revealed an increase from 2 to 4.4 L/min in the inlet flow rate, in addition to an increase in the permeate flux at various concentrations (25, 50, 100, and 150 ppm). The permeate flux increased by 5.9% from 79.71 to 84.38 kg/m^2^ h, noting that the permeate flux improved with increases in the feed flow rate. Figure 5b shows the effects of flow rate on the nickel ion reduction % for different feed concentrations at a constant T = 25 °C and P = 20 bar, exposing that when the inlet flow rate increased from 2 to 4.4 L/min, there was a negligible effect on the nickel ion removal % for all the investigated concentrations (25, 50, 100, and 150 ppm). The observations from Figure 5a,b are matched with those in [24,29].

### 3.5. Factor (TCF) of Temperature Correction

Equation (3) is the governing equation for calculating the temperature correction factor (TCF) as a ratio between the permeate flux variation with temperature:

(3) $$TCF=\frac{Permeate\;flux\;at\;t\;\left(℃\right)}{Permeate\;flux\;at\;25\;\left(℃\right)}$$

Table 2 shows that the system has a higher permeate flux due to the increasing TCF as the temperature increases.

**Figure 6:**
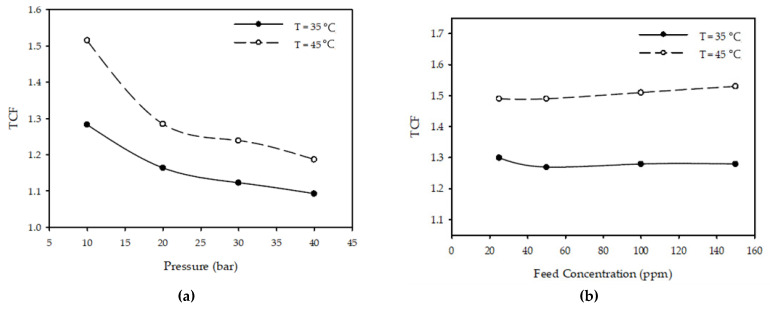

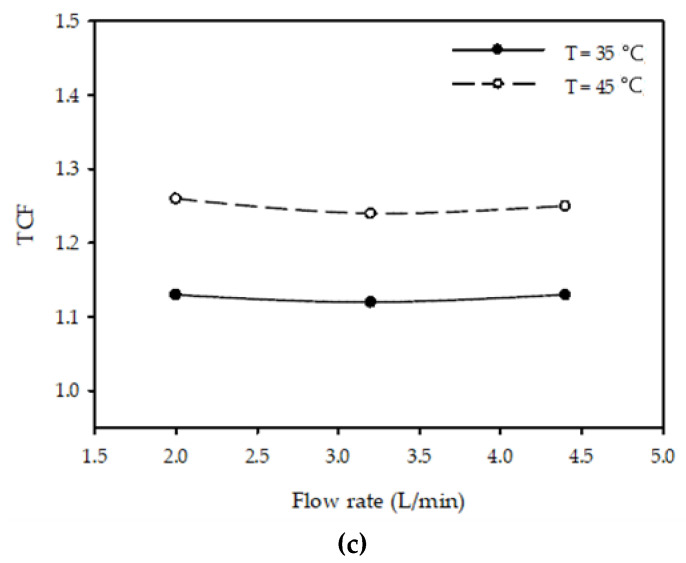
Effect of (**a**) pressure (3.2 L/min, 100 ppm), (**b**) feed concentration (20 bar, 3.2 L/min), and (**c**) flow rate (30 bar, 100 ppm) on TCF at different temperatures.

Figure 6a demonstrates the changes noted to the TCF by altering the operating temperatures of the process while maintaining a constant flow rate at 3.2 L/min with a 100 ppm feed concentration. The results revealed that increasing the pressure from 10 to 40 bar caused a reduction in the TCF values by 17% from 1.28 to 1.09 for a temperature of 35 °C, and a further reduction from 1.52 to 1.19 (around 28%) when the temperature rose to 45 °C.

Figure 6b demonstrates that when the inlet concentration increased from 25 to 150 ppm, the temperature correction factor remained constant at different temperature values for a fixed pressure of 10 bar and a flow rate of 3.2 L/min. Similarly, Figure 6c demonstrates that when the inlet flow rate was raised from 2 to 4.4 L/min, the factor of temperature correction followed the same pattern and remained constant throughout different temperatures while maintaining an applied pressure of 30 bar and an inlet concentration of 100 ppm.

### 3.6. Mathematical Model

Based on the obtained data from the experiments, two mathematical models were proposed. One was designed to predict the obtained permeate flux, and another was designed to predict the nickel removal %. The models included four parameters: concentration, temperature, pressure, and flow.

#### 3.6.1. Mathematical Modeling of Collected Permeate Flux

Initially, it was assumed that the parameters influence the permeate flux independently, and hence, superposition is maintained, i.e.:

(4) $$flux=f\left(T\right)+h\left(C\right)+g\left(P\right)+m\left(F\right)$$

where *f(T), h(C), g(P), and m(F)* are the temperature, concentration, pressure, and flow functions, respectively. Changing one parameter while keeping the others constant produced a similar response, as illustrated in Figure 2a, Figure 3a, Figure 4a, Figure 5a. Changing a single parameter (concentration or flow) resulted in similar, but shifted, curves in these figures. Thus, the relationship between the permeate and every parameter could be modeled independently of the other parameters. By looking at Figure 2a, Figure 3a, Figure 4a, Figure 5a, the equations in Table 3 were developed.

The permeate flux can be modeled by:

(5) $$flux=a_{0}+a_{1}T+a_{2}P+a_{3}P^{2}+a_{4}C+a_{5}C^{2}+a_{6}F$$

where the constants *a*_0_ to *a*_6_ were proposed to reduce the square error between the experimental and modeled data ${\left(flux_{experiment}-flux_{model}\right)}^{2}$. MATLAB® (R2019b, MathWorks, Natic, CA, USA) was used to optimize the constants, which are given in Table 4.

**Figure 7:**
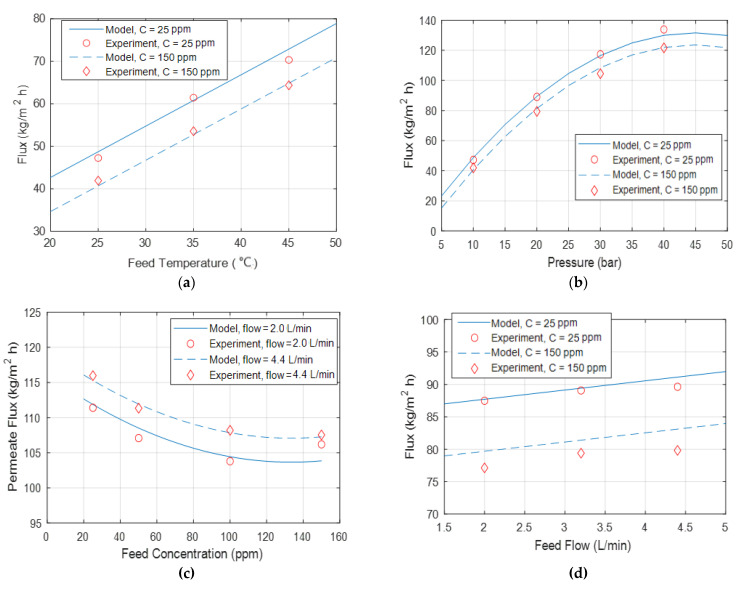
Permeate flux comparative presentation between experimentally obtained and modeled data under different (**a**) temperatures, (**b**) pressures, (**c**) concentrations, and (**d**) flow rates.

Figure 7a–d compares the experimentally obtained data with those generated from the elaborated model for a temperature of 25 °C, a concentration of 25 ppm, a pressure of 20 bar, and a flow rate of 3.2 L/min. It was observed that the model corresponded to the experimental data.

#### 3.6.2. Nickel Rejection Model

In Equation (2), *C_F_* is the only parameter that has to be modeled because *C_p_* is already known. The nickel removal % model was developed using the same approach as the permeate flux model. Table 5 shows the relationship functions.

Thus, the rejection percentage of nickel (*R*) can be modeled as:

(6) $$R=\left(1-\frac{b_{0}+b_{1}T+b_{2}T^{2}+b_{3}P+b_{4}P^{2}+b_{5}P^{3}+b_{6}C+b_{7}C^{2}+b_{8}C^{3}+b_{9}F+b_{10}F^{2}}{C_{F}}\right)\times 100\%$$

where the optimization constants *b*_0_ to *b*_9_ are used to minimize the square error ${\left(R_{data}-R_{model}\right)}^{2}$. Table 6 shows the optimal constants (*b*_0_ to *b*_9_) determined using MATLAB®.

**Figure 8:**
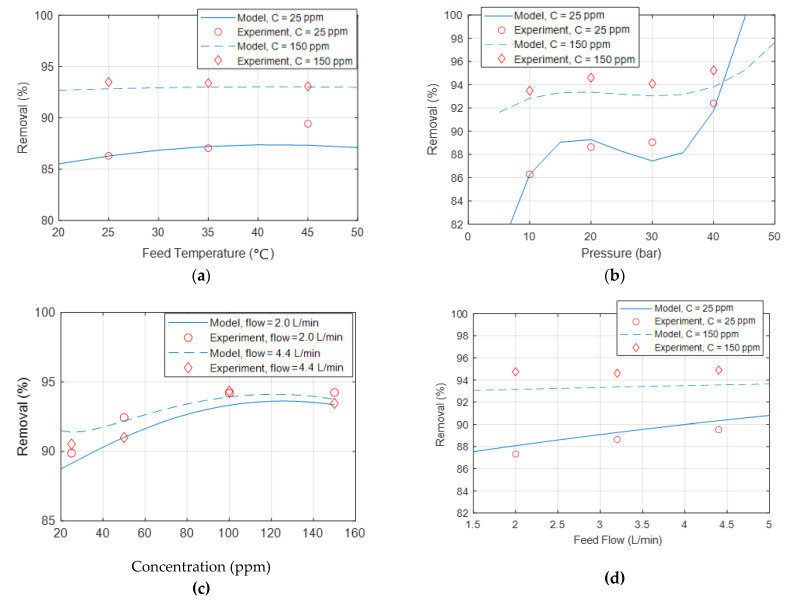
Comparison between nickel rejection percentages obtained experimentally vs. model-generated data under different (**a**) temperatures, (**b**) pressures, (**c**) concentrations, and (**d**) flow rates.

Figure 8a–b show the nickel rejection % comparisons between the experimentally obtained and model-developed data for a temperature of 25 °C, a concentration of 25 ppm, a pressure of 20 bar, and a flow of 3.2 L/min. As can be seen from the figures, the model matched the data collected from the experiments. It should be noted that the coefficient *b*_8_ was very small. This small value increased the error related to the comparison between the results obtained by the developed model and those generated experimentally, especially at low concentrations, since the data collected could not provide readings with such accuracy. Eliminating this coefficient would reduce the fitting function to a quadratic equation, increasing the error between the model data and the experimental data.

## 4. Conclusions

The influence of inlet temperature, inlet concentration, operating pressure, and inlet flow rate on the filtrate flux and nickel ion separation by RO was studied experimentally and mathematically. It was noted that the filtrate flux and Ni (II) ion elimination % were directly proportional to the feed temperature for various feed concentrations. Increasing the temperature from 25 to 45 °C yielded an enhancement of the permeate flux by 49%, owing to changes in the viscosity of the solution and the membrane’s physical properties. Likewise, the permeate flux and Ni (II) ion elimination % were directly proportional to the operating pressure. If the pressure rose from 10 to 40 bar, the permeate flux increased by 188% and the elimination % of Ni (II) ions increased to 95.19%. In contrast, the permeate flux and the percentages of Ni (II) ions removed were inversely proportional to the inlet concentration. On the other hand, the rate of feed flow had a negligible effect on the permeate flux and Ni (II) ion elimination % for various feed concentrations. The temperature correction factor (TCF) was proportional to the temperature, but inversely proportional to the pressure; nevertheless, the inlet concentration and inlet flow rate did not influence the TCF. Developed mathematical models were built for calculating the permeate flux and elimination percentage. It was noted that both models matched the data obtained from the experiments.

## Figures and Tables

**Table 1:** Review of previous investigations on nickel (II) removal by different treatment techniques.

Membrane Type	Initial Conc. (ppm)	Operating Pres. (bar)	pH	Rejection Efficiency %	References
UF	1000	1	3–9	99.1	[16]
UF	50	2	3–9	94–98	[17]
NF	5–250	4–20	2–8	92–98	[19]
NF	60–130	10–30	3.5–10	99.2	[20]
NF	50–200	1–4	2–5.5	85	[21]
NF	5–250	5–20	1–9	98.90	[22]
RO	44–169	11	5.5–7	99.7	[23]
RO	200–600	4–12.5	5–5.5	99.0	[24]
RO	5–500	1–5	3–9	95	[25]
RO	50–150	4–10	-	95.7	[26]
RO	50–200	1–4	2–5.5	98.5	[27]

**Table 2:** Temperature correction factor values with different operating pressures and flow rates, and a feed concentration of 100 ppm.

Flow Rate (L/min)	Pressure (bar)	TCF	
		35 °C	45 °C
2.0	10	1.27	1.54
	20	1.18	1.30
	30	1.13	1.26
	40	1.08	1.18
3.2	10	1.28	1.52
	20	1.16	1.29
	30	1.12	1.24
	40	1.09	1.19
4.4	10	1.30	1.52
	20	1.16	1.28
	30	1.13	1.25
	40	1.10	1.19

**Table 3:** Derived functions relating permeate flux with other parameters.

Permeate Flux vs.	Relationship Function	Justification
Temperature	$f\left(T\right)=aT+b$	Figure 2a shows linear behavior.
Pressure	$g\left(P\right)=aP^{2}+bP+c$	Figure 3a demonstrates linear behavior, but it was not maintained at elevated pressures. Such behavior was balanced by incorporating the term *aP*^2^.
Concentration	$h\left(C\right)=aC^{2}+bC+c$	Figure 4a shows nonlinearity, particularly at lower concentrations. Fitting between the concentration and permeate can be achieved by using a parabolic curve.
Flow	$m\left(F\right)=aF+b$	Figure 5a demonstrates slight differentiation along the permeate flux that can be modeled linearly.

**Table 4:** The optimal values of permeate flux model fitting constants.

Constant	Optimal Value
*a*_0_	−36.1488
*a*_1_	1.2078
*a*_2_	6.1149
*a*_3_	−0.0681
*a*_4_	−0.1867
*a*_5_	0.0007
*a*_6_	1.4248

**Table 5:** Derived functions relating permeate concentration (*C_p_*) with other parameters.

Permeate Flux vs.	Relationship Function	Justification
Temperature	$f\left(T\right)=aT^{2}+bT+c$	Figure 2b demonstrates nonlinear behavior with a similarity to parabolic curves.
Pressure	$g\left(P\right)=aP^{3}+bP^{2}+cP+d$	Figure 3b shows maxima and minima. The use of a quadratic equation would be insufficient for the model due to the singularity values of the minima/maxima; thus, it required a higher-order (cubic) function.
Concentration	$h\left(C\right)=aC^{3}+bC^{2}+cC+d$	Figure 4b, a plot of *C_p_* vs. concentration, yields a curve with more than one minimum; hence, a cubic fitting equation was adopted.
Flow	$m\left(F\right)=aF^{2}+bF+c$	Figure 5b demonstrates a nonlinear pattern with the flow, with one maximum value; hence, a quadratic model was used.

**Table 6:** The optimal values of the nickel rejection model fitting constants.

Constant	Optimal Value
*b*_0_	16.7254
*b*_1_	−0.1830
*b*_2_	0.0022
*b*_3_	−1.6601
*b*_4_	0.0732
*b*_5_	−0.0010
*b*_6_	0.1424
*b*_7_	−0.0006
*b*_8_	1.1594 × 10^−6^
*b*_9_	−0.6872
*b*_10_	0.0274

## Data Availability

The data can be provided upon reasonable request.
